# XTACC3–XMAP215 association reveals an asymmetric interaction promoting microtubule elongation

**DOI:** 10.1038/ncomms6072

**Published:** 2014-09-29

**Authors:** Gulnahar B. Mortuza, Tommaso Cavazza, Maria Flor Garcia-Mayoral, Dario Hermida, Isabel Peset, Juan G. Pedrero, Nekane Merino, Francisco J. Blanco, Jeppe Lyngsø, Marta Bruix, Jan Skov Pedersen, Isabelle Vernos, Guillermo Montoya

**Affiliations:** 1 Structural Biology and Biocomputing Programme, Spanish National Cancer Research Centre (CNIO), Macromolecular Crystallography Group, c/Melchor Fdez. Almagro 3, 28029 Madrid, Spain; 2 Macromolecular Crystallography Group, Novo Nordisk Foundation Center for Protein Research, Faculty of Health and Medical Sciences, University of Copenhagen, Blegdamsvej 3B, 2200 Copenhagen, Denmark; 3 Centre for Genomic Regulation (CRG), Dr Aiguader 88, 08003 Barcelona, Spain; 4 Universitat Pompeu Fabra (UPF), 08003 Barcelona, Spain; 5 Department of Biological Physical Chemistry, Spanish National Research Council (CSIC), Institute of Physical Chemistry ‘Rocasolano’, Serrano 119, 28006 Madrid, Spain; 6 Structural Biology Unit, CIC bioGUNE, 48160 Derio, Spain; 7 IKERBASQUE, Basque Foundation for Science, 48013 Bilbao, Spain; 8 Interdisciplinary Nanoscience Center (iNANO) and Department of Chemistry Aarhus University, Gustav Wieds Vej 14, Building 1590-252, 8000 Aarhus C, Denmark; 9 Institució Catalana de Recerca i Estudis Avançats (ICREA), Pg. Lluís Companys 23, 08010 Barcelona, Spain

## Abstract

chTOG is a conserved microtubule polymerase that catalyses the addition of tubulin dimers to promote microtubule growth. chTOG interacts with TACC3, a member of the transforming acidic coiled-coil (TACC) family. Here we analyse their association using the *Xenopus* homologues, XTACC3 (TACC3) and XMAP215 (chTOG), dissecting the mechanism by which their interaction promotes microtubule elongation during spindle assembly. Using SAXS, we show that the TACC domain (TD) is an elongated structure that mediates the interaction with the C terminus of XMAP215. Our data suggest that one TD and two XMAP215 molecules associate to form a four-helix coiled-coil complex. A hybrid methods approach was used to define the precise regions of the TACC heptad repeat and the XMAP215 C terminus required for assembly and functioning of the complex. We show that XTACC3 can induce the recruitment of larger amounts of XMAP215 by increasing its local concentration, thereby promoting efficient microtubule elongation during mitosis.

## Abstract (web summary)

chTOG, a microtubule polymerase, interacts with TACC3 during mitosis to regulate spindle formation. By studying their *Xenopus* homologues, Mortuza *et al*. discover that one TACC3 recruits two chTOG molecules to the spindle, increasing its local concentration and promoting microtubule elongation.

## Graphical abstract

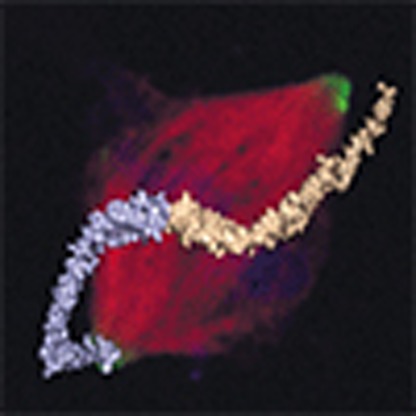

The mitotic spindle is a sophisticated apparatus that assembles during cell division to distribute the genetic material to daughter cells with precision and reliability. Spindle assembly involves a tight spatial and temporal control on motor and non-motor microtubule-associated proteins (MAPs) that collectively define the precise dynamic properties of the microtubules (MTs) and their organization. As a result, the spindle is a robust structure that can exert the forces required to align and segregate the chromosomes while maintaining its highly dynamic nature important for self-correction mechanisms. Spindle defects arising from failures in setting up the right MT properties may lead to chromosomal instability and aneuploidy, one of the most common causes that escort the loss of control over cell growth and may contribute to tumour development^1^.

The widely conserved chTOG/XMAP215 protein family consists of MAPs that promote MT growth and have an essential role in defining mitotic MT dynamics and spindle assembly. These proteins share a C-terminal coiled-coil domain and an N-terminal region composed of different numbers of TOG domains, each binding one soluble tubulin dimer^2^,^3^. *In vitro* experiments have shown that XMAP215 acts as a processive polymerase, catalysing the addition of ~25 tubulin dimers at the growing MT plus ends^3^. Recently, it was shown that XMAP215 polymerizing activity directly defines mitotic spindle length^4^. The precise control on chTOG/XMAP215 activity during mitosis is therefore utterly important for the assembly of the bipolar spindle. However, without detailed structural insight, it is difficult to understand how this control could be exerted.

Although chTOG/XMAP215 proteins bind directly to the MTs *in vitro*, their binding affinity can be modulated as it has been shown to increase through a conserved interaction with TACC3 (transforming acidic coiled-coil 3; refs 5, 6, 7, 8). TACC proteins were first identified in a search of genomic regions that are amplified in breast cancer^9^. Subsequently, TACC proteins were described in different organisms, ranging from yeasts to mammals. Functional studies in different systems have pointed to a conserved function of TACC3 in promoting MT assembly in mitosis through its interaction with chTOG/XMAP215 (refs 7, 8, 10). Indeed, reducing TACC3 protein levels impairs the correct localization of chTOG/XMAP125 to the mitotic centrosome^11^,^12^,^13^, and increasing the concentration of TACC3 results in the enhanced recruitment of chTOG/XMAP215 to the spindle poles^7^,^8^,^9^. By contrast, chTOG/XMAP215 is not required for the localization of TACC3 proteins^5^,^14^. Although TACC3 phosphorylation by Aurora A during mitosis is essential for its function, it does not regulate its interaction with chTOG/XMAP215 (ref. 8), and consistently it does not enhance the MT binding affinity of the TACC3–chTOG complex. However, the molecular mechanism by which TACC3 enhances XMAP215 recruitment to MTs and spindle poles is not well understood^8^,^13^.

To fully understand the functional implications of XTACC3–XMAP215 interaction and the regulation of MT dynamics during mitosis, we have used a hybrid approach to dissect key interactions. Here we define the minimal active TACC domain (TD) of XTACC3 and derive a structural model by small-angle X-ray scattering (SAXS). We identify key residues in the TD and the C terminus of XMAP215 required for the interaction, and through site-directed mutagenesis we test the consequences of specifically disrupting it for spindle assembly. Our data reveal a mechanism by which XTACC3 recruits XMAP215, and thereby promotes efficient MT elongation in mitosis. Based on our data, we propose a model for the regulatory role of TACC3 on XMAP215/chTOG activity during cell division. This mechanism connecting the C-terminal coiled-coil domains of XMAP215 and XTACC3 could be extended to their mammalian homologues.

## Results

### XMAP-Ct and TD4 are the minimal interacting domains

**Figure 1:**
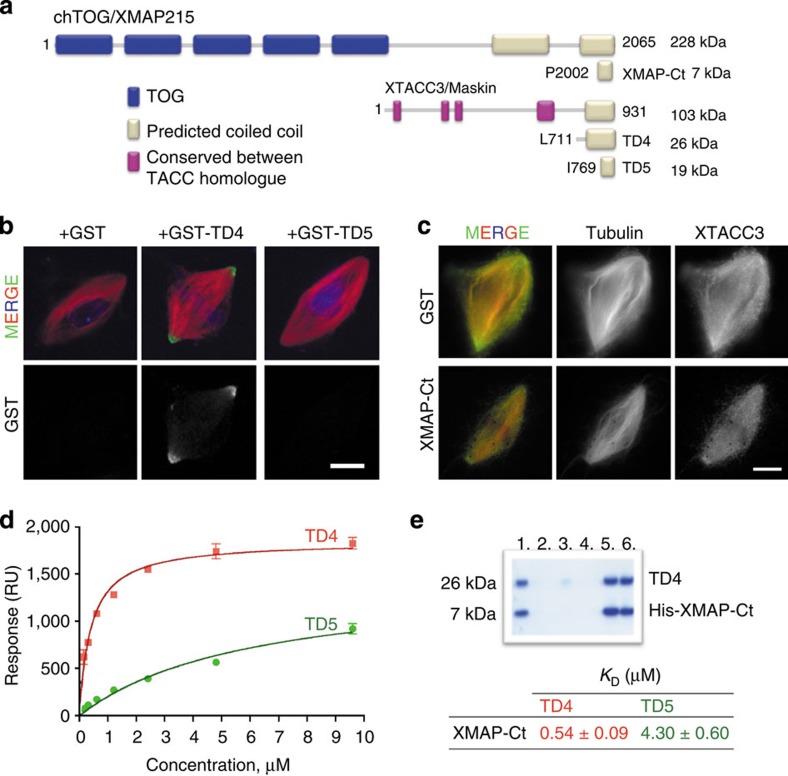
Defining the minimal XMAP215 and XTACC3 binding domain and its effect on localization to the spindle poles and assembly. (**a**) The chTOG/XMAP215 and XTACC3/Maskin domain architecture. Various C-terminal fragments of XMAP215 and XTACC3 were cloned and expressed for biophysical and functional analysis. (**b**) Representative images of spindles assembled in egg extracts containing GST, GST-TD4 and GST-TD5 at 150 nM. Samples were processed for immunofluorescence with an anti-GST antibody. GST-TD4 localized to the spindle poles, whereas GST-TD5 did not. (**c**) Representative images of spindles assembled in egg extracts containing GST or XMAP-Ct (15 μM). Samples were processed for immunofluorescence with anti-XTACC3 antibodies. Bipolar spindles also assemble in egg extracts containing XMAP-Ct, but their tubulin density and XTACC3 localization were reduced compared with controls. Images were taken with identical camera settings. In all cases, DNA is in blue, tubulin in red and GST in green. Scale bars, 10 μm (**b**,**c**). The experiment was repeated four times. (**d**) The interaction between TDs (TD4 and TD5) and XMAP-Ct was tested by SPR, and *K*_D_ values were measured to show that XMAP-Ct has different binding affinities for TD4 and TD5. (**e**) SDS–PAGE showing pull-downs of TD4 using His-XMAP-Ct. Lane 1: reaction mixture; lanes 2–4: washes and lanes 5–6, elution. RU, response unit.

To define the structure and function of the XTACC3–XMAP215 complex, various C-terminal TACC and XMAP215 fragments were designed using different prediction software and controlled proteolysis. The corresponding constructs were prepared and the recombinant proteins were expressed, purified and verified by mass spectrometry (Fig. 1a; Supplementary Fig. 1a and Supplementary Table 1, which contains the names used through the manuscript and describes all the proteins, peptides and mutants in this manuscript).

The TD has been shown to mediate the interaction of XTACC3 with XMAP215 (refs 8, 13). Five TDs (only data for 4 and 5 are shown) were characterized by circular dichroism (CD) showing secondary structural elements predominantly helical with two distinct unfolding transitions exhibited by TD4, whereas TD5 depicted only a single transition (Supplementary Fig. 1b), suggesting that these proteins have different stability and tertiary fold. The TDs oligomerization behaviour^9^ was validated by electron microscopy to show that they form long filaments (Supplementary Fig. 1c). These assemblies had a tendency to oligomerize in one direction, generating protofilaments of different lengths and were generally irregular, occasionally forming sheets built up by several long filaments.

To further characterize the TDs, we used the *Xenopus* egg extract as previously described^8^,^13^. Spindles were assembled in cycled egg extracts supplemented with the different GST-TD proteins at XTACC3 endogenous concentrations. Immunofluorescence analysis showed that TD4 not only localized to the spindle poles but also was weakly present along the spindle MTs as previously described for the TD, whereas TD5 did not localize at all (Fig. 1b,c and Supplementary Fig. 2). Hence, these data show that TD4 is the minimal TD of XTACC3 that retains both the spindle pole localization and XMAP215 interaction.

Several chTOG homologues have been reported to interact with TACC proteins^5^,^11^,^13^. However, the exact region of the chTOG proteins responsible for this interaction had not been previously described. XMAP215 is a 2065 residues protein composed of five TOG domains and a C-terminal region (Fig. 1a). We excluded TOG domains because they are well known to be involved in tubulin binding^2^, therefore to dissect the minimal XMAP215 region that interacts with XTACC3, different C-terminal fragments of XMAP215 were examined. Within the C-terminal region, two segments are predicted to be coiled coil^15^, however, only one of these domains (XCC3; Supplementary Fig. 1a), hereafter XMAP-Ct, was soluble and could be purified for binding experiments. *In vitro* pull-down and surface plasmon resonance (SPR) measurements indicated that TD4 indeed interacts with XMAP-Ct (Fig. 1d,e). To further validate the interaction between these minimal domains of XTACC3 and XMAP215, SPR experiments were performed wherein XMAP-Ct was attached to a CM5 chip. Consistently, we found that TD4 had a 10-fold increased affinity for XMAP-Ct compared with TD5 (Fig. 1d).

XMAP-Ct is a monomer with almost no secondary structural elements (Supplementary Fig. 3). To obtain additional evidence for the interaction between XMAP-Ct and the minimal XTACC3 binding domain, we tested its ability to compete out the interaction between XMAP215 and XTACC3 in egg extract. Previous experiments have shown that spindles assembled in XTACC3-depleted extract are shorter by 20% and contain 40% less MTs^8^. Consistently, spindles assembled in extract containing XMAP-Ct showed a 20% reduction of tubulin density without affecting the spindle length (Supplementary Fig. 2b). These results suggested that XMAP-Ct indeed interferes with the XTACC3–XMAP215 interaction, thereby reducing the level of MT polymerization. Altogether, our data show that TD4 and XMAP-Ct are indeed the minimal interacting domains.

### XMAP215 and XTACC3 interact through a coiled-coil assembly

**Figure 2:**
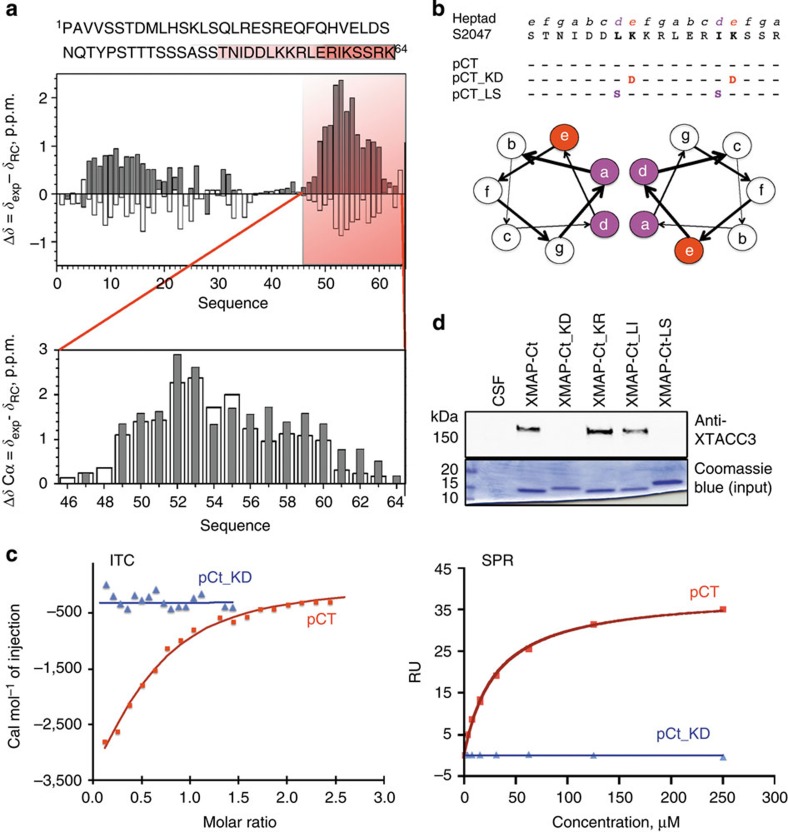
C-terminal binding region of XMAP-Ct is a bona fide coiled coil. (**a**) Conformational chemical shift pattern by NMR of XMAP-Ct (pH 5.9 and 25 °C) as a function of the protein sequence suggests a helical propensity. Above: Cα (grey bars) and Cβ (white bars); below: comparison of the Cα conformational chemical shits of XMAP-Ct (white bars) and pCt (pH 6.1, 5 °C; grey bars). (**b**) A peptide derived from XMAP-Ct coiled-coil region was synthesized (pCt). Positions e and d of the heptad repeat were mutated to introduce either an opposite charge (pCt KD) or to break the hydrophobic interaction (pCt_LS). Scheme of a wheel diagram depicting the disposition of key residues in a simple two-helix coiled-coil interaction. A coiled-coil interaction of a more complex nature (trimer/tetramer) will also follow the same principle forming a hydrophobic core with the charged residues pointing outwards. (**c**) ITC and SPR binding curves showing TD4 interaction with pCt (red) and no interaction with pCt_KD (blue). The calculated *K*_D_ values were 11 and 30 μM by ITC and SPR, respectively. (**d**) Western blot of pull-downs of CSF, XMAP-Ct and its mutants. Only XMAP-Ct and mutants of conserved charge (XMAP-Ct_KR and XMAP-Ct_LI) can pull down XTACC3, whereas by reversing the charge (XMAP-Ct_KD and XMAP-Ct_LS) mutants were unable to pull down XTACC3. The efficiency of the pull-down of the tagged proteins was verified by Coomassie blue staining. RU, response unit.

Having defined the minimal domains involved in the XTACC3–XMAP215 interaction, nuclear magnetic resonance (NMR) spectroscopy was used to characterize the XMAP-Ct binding site. The complete NMR assignment of the XMAP-Ct backbone atoms revealed that it does not adopt a globular fold (Supplementary Fig. 4a). Most of the Cα variations (with respect to the random coil values) are positive and the Cβ negative (Fig. 2a) indicating a tendency to adopt a helical conformation (12%). Two regions showed more populated helical content, corresponding to XMAP-Ct residues 5–20 (23%) and 46–61 (38%) (XMAP215 numbering 2006–2021 and 2047–2062, respectively). Using a synthetic peptide (pCt) corresponding to this second region (residues 46–61) (Fig. 2b and Supplementary Table 1), we found that the C-terminal part of the sequence was further stabilized by local interactions. pCt showed a similar helical propensity (49%) in the absence of the N-terminal part of the XMAP-Ct domain sequence (Fig. 2a) and a good binding affinity to TD4 (Supplementary Fig. 5).

The successful complete assignment of XMAP-Ct was then used to follow its interaction with TD4, using two different approaches. First, changes in the ^15^N-HSQC (heteronuclear single quantum correlation) signals of free XMAP-Ct in the presence of increasing amounts of unlabelled TD4 were monitored (Supplementary Fig. 4b). After the addition of TD4, most of the signals from the interacting region at the C-terminal (L52, K53, K54, L55, E57, R58 and I59) became broader (probably owing to exchange processes with folded structures) and disappeared from the spectra. Second, by measuring the interaction between TD4 and pCt (Supplementary Table 1). This was followed by monitoring changes in the position and line width of TD4 resonances in ^15^N-HSQC spectra recorded in the presence and absence of the peptide (Supplementary Fig. 4c). Upon complex formation, a set of signals shifted and others disappeared corroborating a direct interaction (Supplementary Fig. 4c). In order to map the association surface of TD4 with pCt, we were able to assign a subset of resonances spanning the TD4 sequence. The analysis revealed that TD4 residues S4, S71 and A78 do not participate in binding, and many residues within the A159–I219 segment are implicated in the interaction with pCt (Supplementary Fig. 4c, inset), supporting the participation of the final TD helix (α4) in the recognition of the XMAP-Ct peptide.

Two techniques, isothermal titration calorimetry (ITC) and SPR measurements, independently confirmed the binding of pCt with TD4 (Fig. 2c). Moreover, the ITC measurements indicated a TD4:pCt stoichiometry of 0.5, suggesting that one TD4 can bind two pCt molecules (Supplementary Fig. 5a). To further characterize binding, we performed gel filtration experiments to observe whether the peptide elution was shifted in the presence of TD4 (Supplementary Fig. 5b). Given the concentration-dependent self-association behaviour of the TDs, the oligomerization state of TD4 was examined by multiple-angle light scattering (MALS), confirming that TD4 at 1.5 mg ml^−1^ is a dimer (Supplementary Fig. 5c). Although lower concentrations were tested, TD4 still behaved as a dimer. The TD4:pCt peak was also analysed by MALS, showing that the domain in the presence of saturating concentrations of pCt displays a mass of 65.4 kDa (Supplementary Fig. 5c), suggesting that four molecules of the peptide can bind the TD4 dimer in agreement with the ITC analysis.

XMAP-Ct is predicted to be a coiled-coil domain and therefore pCt should follow a signature of heptad repeats whereby there are hydrophobic groups at positions a and d, charged residues at positions b and e, and sideways interactions occur between positions b, e and f (see the scheme in Fig. 2b)^16^. Various XMAP-Ct peptide mutants were strategically designed following this rationale and were synthesized to disrupt the heptads either by introducing an opposite charge mutation at position e, that is, changing K to D (pCt_KD) or by disturbing the hydrophobic interactions by adding a nucleophilic head group at position d, that is, changing L to S (pCt_LS) (Fig. 2b and Supplementary Table 1). As expected, pCt_KD and pCt_LS are critical point mutations that disrupt the heptad repeat and abolished any XMAP-Ct–TD interaction *in vitro* (Fig. 2c). To test whether these mutations could also be sufficient to impair the interaction between XMAP-Ct and XTACC3 in *Xenopus* egg extract, site-directed mutagenesis was performed on XMAP-Ct to generate XMAP-Ct-KD and XMAP-Ct-LS (Supplementary Table 1). In addition, two new conservative mutations were introduced as controls generating XMAP-Ct-KR and XMAP-Ct-LI. In contrast to XMAP-Ct, neither XMAP-Ct-KD nor XMAP-Ct-LS could pull down XTACC3 from the egg extract. However, the XMAP-Ct proteins bearing conservative changes did efficiently pull down XTACC3 (Fig. 2d). Hence, our results show that XMAP-Ct contains a bona fide coiled coil at its C-terminal region that is necessary for XTACC3 binding.

### SAXS structure of a TD

**Figure 3:**
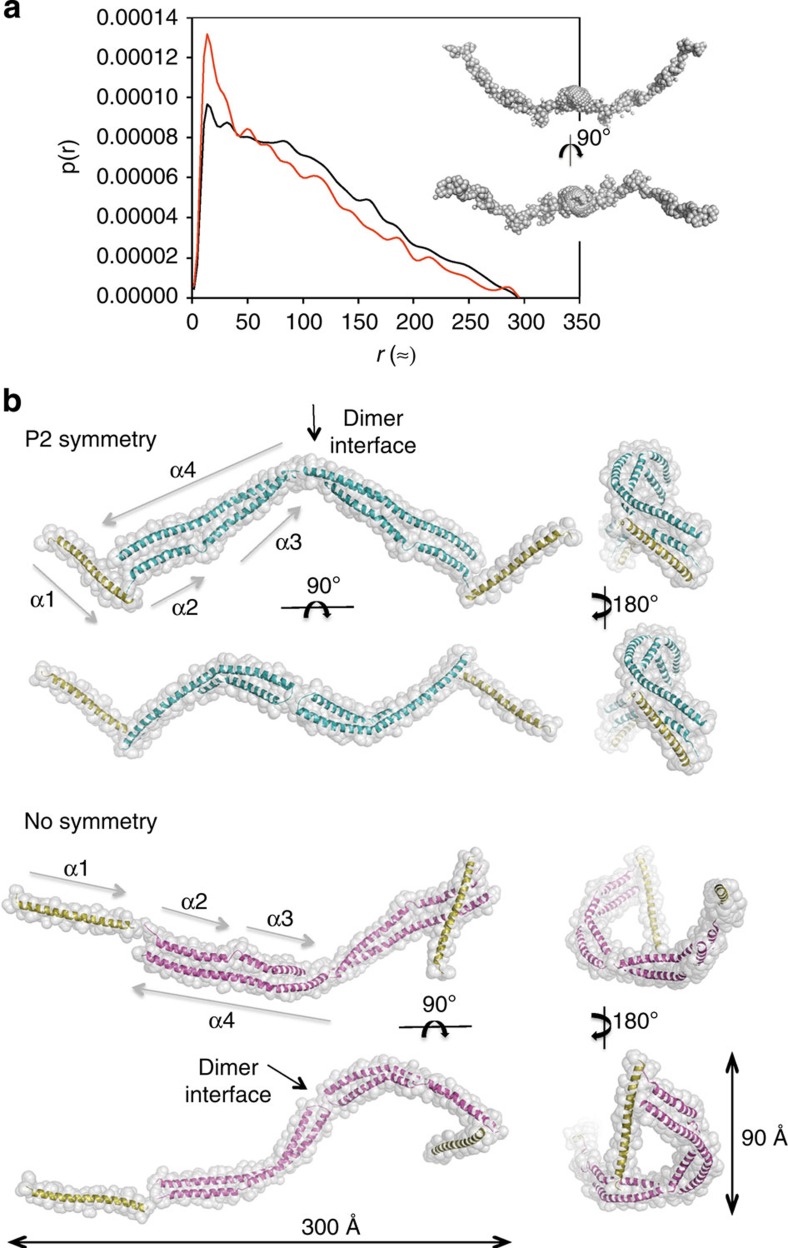
SAXS measurements and structure models of TD4. (**a**) Pair distance distribution function *p*(*r*) for TD4 alone (black) or with pCt (red) are shown together in an *ab initio* GASBOR^34^ model. (**b**) A TD4 dimer model with and without P2 symmetry is composed of four helices (α1–α4). The models were manually constructed and were optimized using CORAL^38^. The two models have also been turned 90° around two perpendicular axes to show different projections.

The high flexibility and oligomerization propensity of the TD makes it a difficult target for structure determination by NMR or crystallography. An alternative approach to obtain structural insights on this type of polypeptides is the use of SAXS, generating models that can be further validated by biochemical and functional assays. All experiments to derive our model (Fig. 3a) were carried out at 1.5 mg ml^−1^, where TD4 behaved as a single species (Supplementary Fig. 5c). SAXS analysis was performed on TD4, TD4:pCt and a TD4 mutant (TD4-M2), to show that mutations used in functional assays did not affect the global structure (Supplementary Table 2 and Supplementary Fig. 7). An initial model of the TD4 monomer was obtained using I-Tasser^17^. The model was further optimized using rigid body refinement obtaining a good fit. Initially, no symmetry was imposed in the calculations; however, further runs were performed applying a P2 symmetry. In both cases, with or without symmetry, the two models fitted the data very well with similar quality. Thus, the SAXS data does not distinguish between the two TD4 models (Fig. 3b). The results for the molecular mass were derived from the forward scattering, and the radius of gyration are displayed in Supplementary Table 2.

The final models indicate that TD4 is an elongated dimer with an interface between the loop region of helices α3 and α4, the junction of helices of α2 and α3 interacting with the long helix (α4) at the central part of the molecule and a single helix (α1) extending outwards. This fits well with the molecular dimensions (300 × 90Å) of the two models, with or without P2 symmetry (Fig. 3b). Furthermore, the elongated dimer model with no applied symmetry shows α1 orientated in different directions, suggesting a possible flexible helical arm. The model also suggests that the dimer interface lies between the loop regions of helices α3–α4 with one molecule rotated by 180° (in the *x* axis) with respect to the other. This configuration could aid to stabilize the dimer through a summation of electrostatic and hydrophobic interactions attracting the two molecules (Supplementary Fig. 6).

The thermal denaturation of TD5, which has a deletion of α1 and lacks the first unfolding transition displayed by TD4 (Supplementary Fig. 1b), suggest that helix α1 may have a role in stabilizing the tertiary structure. Remarkably, although TD5 did not localize to the spindle poles (Fig. 1b,c), *in vitro* SPR experiments showed that it interacts with XMAP-Ct but with a lower affinity (Fig. 1d). This suggests that the lack of localization is possibly a consequence of protein stability rather than a loss of XMAP215 binding.

### The TD contains key residues for XMAP215 binding

**Figure 4:**
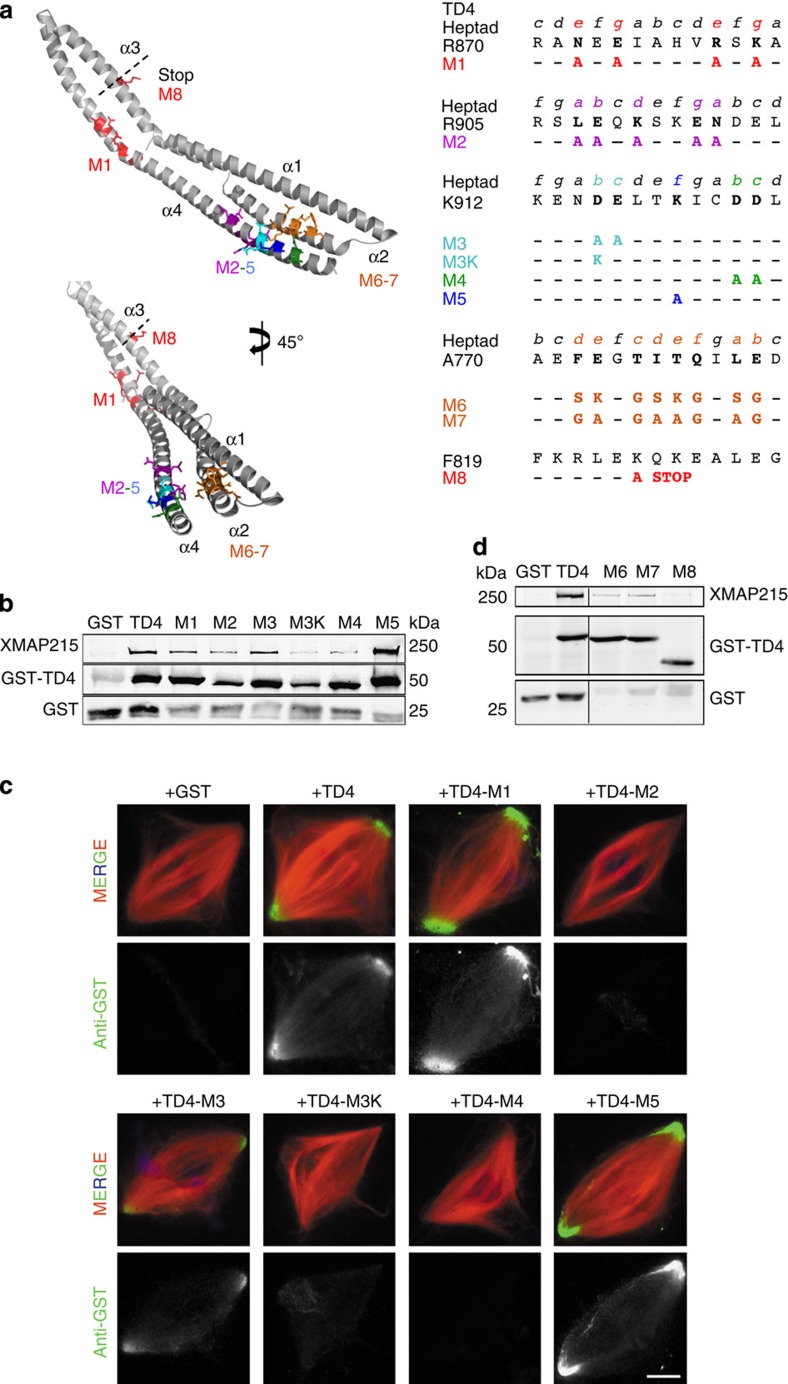
Mapping the XMAP-Ct binding site on TD4. (**a**) SAXS structure of a TD4 monomer highlighting regions where mutations were performed in stick representation. Two regions on α4 were detected by NMR to change upon XMAP-Ct binding. (**b**) Western blot analysis of anti-GST pull-downs from egg extracts containing various GST fusion proteins corresponding to the different mutations on α4 of TD4. The blots were examined with anti-XMAP215 (upper lane) and anti-GST (middle and bottom lanes). GST-TD4, TD4-M1, TD4-M3 and TD4-M5 can pull down endogenous XMAP215. GST-TD4-M2 can also pull down XMAP215 but less efficiently. However, the mutants affecting the polarity of this heptad region (TD4-M3K and M4) strongly abrogated XMAP215–TD4 interaction. (**c**) Representative images of spindles assembled in egg extracts containing GST or the different GST-TD4 mutants as indicated. The samples were processed for immunofluorescence using the anti-GST antibody. Images were taken with identical camera settings. GST-TD4, TD4-M1, TD4-M3 and TD4-M5 localize at the spindle poles, whereas GST, -M2, -M3K and -M4 do not. DNA is in blue, tubulin in red and GST in green. Scale bar, 10 μm. (**d**) Western blot analysis of anti-GST pull-downs from egg extracts containing various GST fusion proteins corresponding to the different mutations on α2 and α3 of TD4, all of which affected the XMAP215–TD4 interaction. The blots were examined with anti-XMAP215 (upper lane) and anti-GST (middle and bottom lanes).

NMR analysis of TD4–pCt identified two regions on the TD that display a shift or loss of signal intensity implying significantly perturbed residues upon protein–protein interaction (Supplementary Fig. 4). To further assess the importance of these regions, they were located onto the SAXS structures for additional evaluation (Fig. 4a). The two regions of interest (ROIs) were on α4, one of which is in the middle of the helix, whereas the second is within the last 20 amino-acid residues. Alanine-scanning mutations, including strategic positions on the heptad repeat, were performed along these sections (Fig. 4a). The corresponding GST fusion proteins were expressed and purified to perform pull-downs and localization studies in egg extracts (Supplementary Fig. 8 and Supplementary Table 1). TD4-M1, TD4-M3 and TD4-M5 pulled down endogenous XMAP215 (Fig. 4b); however, TD4-M2 showed a low binding affinity, which was further reduced for TD4-M4. Although TD4-M3 did pull down XMAP215, reversing the amino-acid polarity by changing D to K (TD4-M3K) completely abolished the interaction. The localization of these mutants to spindles was examined by adding the recombinant proteins to egg extracts at XTACC3 endogenous concentrations. Immunofluorescence analysis showed that all the proteins that retained an interaction with XMAP215 (TD4-M1, TD4-M3 and TD4-M5) localized to the spindle poles in a similar manner to the original TD4 protein (Fig. 4c). In contrast, those having reduced binding to XMAP215 (TD4-M2, TD4-M4 and TD4-M3K) did not localize.

A recent study mapped the interaction of TACC3 to a different region of chTOG^18^. The authors proposed that the chTOG–TACC3 interaction involves a putative sixth TOG domain and the adjacent C-terminal section (residues 1517–1957, chTOG numbering). Although this region includes part of the N-terminal residues of XMAP-Ct (2002–2049, XMAP215 numbering) it does not include the key residues for the association identified in our study. Consistently, the affinity for the interaction between the TD and this fragment of chTOG^18^ is much lower than the affinity we found for an equivalent TD (TD4) and XMAP-Ct (Fig. 1d,e). Furthermore, the authors proposed that the chTOG binding site on TACC3 is located within the ‘triple amino-acid stutter region’ that disrupts the heptad repeats. Deletion mutants within this region showed chTOG binding abrogation. We therefore mutated positions *a, d* and *e* of the putative heptad repeat by either adding a charged residue (TD4-M6) or keeping it neutral (TD4-M7) to examine whether this section is indeed important for XMAP215 binding (Fig. 4d). These mutants inefficiently pulled down XMAP215 from the egg extract, although binding was not completely abrogated. Our SAXS structure provides a structural basis for the requirement of this region in α2 for the interaction as it suggests that the α2 and α4 are stacked onto one another thereby stabilizing the TD structure. This suggests that the deletion of these amino acids in the human TACC3 fragment affects its internal organization, destabilizing the protein, thereby preventing its interaction with the N-terminal region of XMAP-Ct contained in the chTOG fragment (1517–1957).

We conclude that although the N-terminal region of XMAP-Ct may have a stabilizing role in this interaction, the critical TACC binding site in XMAP215 is located in the C-terminal heptad repeats involved in a coiled-coil association (Fig. 4). This is in agreement with the results obtained for mouse TACC3 that was shown to pull down the C-terminal part of chTOG^19^. However, in this work, the authors reported that the chTOG binding site was along α2 (based on our model) of TD. We therefore tested this idea in our experimental system by adding a stop codon on TD4 (M8) to generate an equivalent fragment. We found that this truncated fragment lacking the α4 critical residues was unable to pull down XMAP215 in *Xenopus* extracts (Fig. 4d).

### XTACC3 recruits XMAP215 via a coiled-coil association

**Figure 5:**
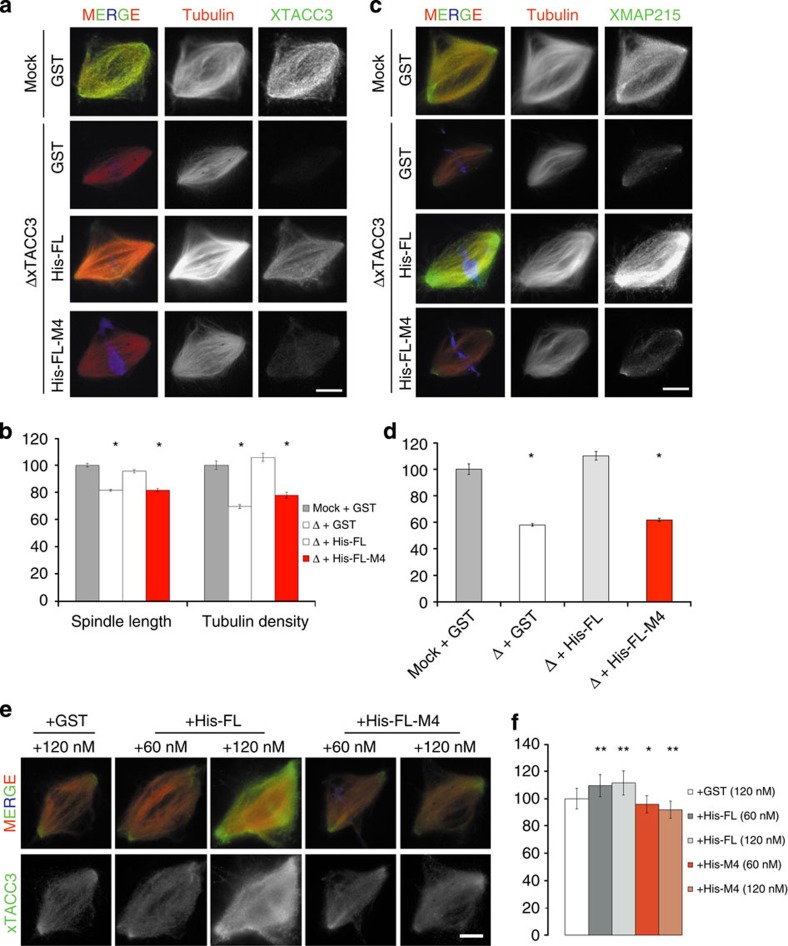
Asp922 and Asp923 are key TD4 residues on α4 for XTACC3–XMAP215 interaction and function. (**a**) Representative images of spindles assembled in mock or XTACC3-depleted extract (Δ) containing GST, His-FL or His-FL-M4 as indicated. Samples were processed for immunofluorescence with anti-XTACC3 antibodies. His-FL-M4 did not localize to the spindle. (**b**) Quantification chart of spindle length and tubulin density of the different samples. Spindles assembled in XTACC3-depleted extracts (Δ) are 20% shorter, and their tubulin density is reduced by 30% compared with controls as previously described. Addition of His-FL to the depleted extract fully restored these parameters unlike that with His-FL-M4. Error bars represent the s.e.m. *P value <0.01, obtained applying a homoscedastic *t*-test. (**c**) Representative images of spindles assembled in mock or XTACC3-depleted extract (Δ) containing GST, His-FL or His-FL-M4 as indicated. Samples were processed for immunofluorescence with anti-XMAP215 antibodies. In XTACC3-depleted extracts supplemented with GST or His-FL-M4, XMAP215 localization to the spindle is strongly reduced. (**d**) Quantifications chart of XMAP215 localization normalized on tubulin density of the different samples. All values are the weighted mean of three independent experiments. Error bars represent the s.e.m. Spindles assembled in XTACC3-depleted extracts (Δ) supplemented with GST or His-FL-M4 display 40% less XMAP215 than controls. *P value <0.01, obtained applying a homoscedastic *t*-test. (**e**) Representative images of spindles assembled in extract containing GST, His-FL, His-FL-M4 as indicated. Samples were processed for immunofluorescence using the anti-XTACC3 antibody. (**f**) Chart showing the spindle length of the different assays. Spindles assembled in extracts supplemented with extra His-FL are longer than those assembled in extract containing GST or His-FL-M4. Error bars represent the s.e.m. *P* values ‘*’and ‘**’ are <0.05 and <0.01, respectively, obtained applying a homoscedastic *t*-test. In all cases, images were taken with identical camera settings. DNA is in blue, tubulin in red and XMAP215 in green. Scale bars, 10 μm.

Having mapped precisely the interaction between XTACC3 and XMAP215, the functional implications of this association for spindle assembly were addressed using the egg extract system. Site-directed mutagenesis was used to introduce the changes equivalent to the TD-M4 mutations (D922A and D923A) in the XTACC3 full-length protein (His-FL-M4). Spindles were assembled in XTACC3-depleted egg extracts supplemented with GST, His-FL or His-FL-M4. The quantification of spindle length and MT density in three independent experiments showed that His-FL-M4 could not rescue the depletion phenotype (Fig. 5a,b). Indeed, the spindles assembled in XTACC3-depleted extract containing His-FL-M4 mutant XTACC3 displayed reduced levels of associated XMAP215 (Fig. 5c,d). Moreover, His-FL-M4 spindle localization was also strongly reduced compared with His-FL or the endogenous XTACC3, showing a weak signal along the spindle MT with a slight enrichment at the spindle poles (Fig. 5a). These results showed that two single-point mutations in XTACC3 (His-FL-M4) were sufficient to impair its binding to XMAP215 and thereby its efficient recruitment to the mitotic MTs. The relevance of XMAP215 recruitment by XTACC3 during spindle assembly was further analysed by evaluating its putative effect on spindle length. The addition of XTACC3 (His-FL) in egg extract resulted in an increase of the spindle length according to the total level of protein (Fig. 5e,f and Supplementary Fig. 8c). In contrast, adding His-FL-M4 to similar levels had no influence on spindle length.

These results provide strong support for the notion that XTACC3 is a key factor regulating the activity of XMAP215 by efficiently forming a C-terminal four-helix coiled-coil assembly, and thereby promote MT growth and spindle assembly.

## Discussion

Many centrosomal and cytoskeleton related proteins are composed of predominantly extended coiled-coil scaffolds that serve as docking sites for regulatory macromolecules. Understanding the mechanism underlying the formation of specific interactions among these coiled-coil proteins is key for the regulation of MT dynamics and organization. However, the precise mechanisms have been difficult to address particularly because they are large flexible coiled-coil proteins and their interactions are transient in nature, further complicating the capture of these entities.

Here we have focused on deciphering a key molecular interaction between two important regulators of MT assembly in dividing cells, TACC3 and chTOG. The function of chTOG has been extensively studied. This MAP has been characterized as a MT polymerase that binds tubulin dimers through its N-terminal TOG domains and brings them to the growing plus ends catalysing their incorporation into the growing tube^3^. During mitosis, chTOG activity has been shown to be key for MT assembly and dynamics by counteracting the activity of the MT depolymerase MCAK^20^. In contrast, the molecular mechanism driving the function of TACC3 in dividing cells has not been precisely established. However, it has been shown in various systems to also promote MT assembly, in particular, at the centrosome in an indirect manner through an interaction with XMAP215.

Using a hybrid approach, we demonstrated that this interaction involves the C-termini of XTACC3 and XMAP215, whereby helices α2 and α4 on XTACC3 contain key residues for XMAP215 complex formation. The TD has a potential to oligomerize whilst XMAP-Ct, predicted to be a coiled coil, appears to be predominantly disordered in the absence of the TD but becomes partially ordered upon complex formation (Supplementary Fig. 4). Attempts to break the dimerization resulted in insoluble or unstable protein. For such an elongated coiled-coil structure, dimerization may be important for stabilizing its fold. Moreover, our SAXS model indicated that α2 and α4 are in close proximity, and thereby of important internal organization further stabilizing the protein fold (Fig. 3). Although recent studies indicate that only one region on TD participate in chTOG interaction (α2 helix)^18^,^19^, our study shows that not only α2 is involved in the association but also that α4 is indeed required for XMAP215 binding (Fig. 4).

Mapping the XTACC3–XMAP215 interaction has allowed us to precisely address its functional relevance for spindle assembly. We showed that changing key residues in the C-terminal heptad of XTACC3 was sufficient to preclude its interaction with XMAP215 in egg extract (Fig. 4). Interestingly, this protein was unable to rescue the spindle phenotypes described for XTACC3 depletion^8^. These findings demonstrate that in this system, the function of XTACC3 is entirely dependent on its interaction with XMAP215, indicating that the tubulin polymerase activity is regulated by XTACC3 during cell division. Furthermore, residues identified in the α4 termini of TD (Asp922 and Asp923) in its interaction with XMAP215 were also described in its interaction with ARNT PAS-B to TACC3, which is essential for the transcriptional response to hypoxia inducible factor^21^. A sequence alignment including different species and isoforms indicates that these residues are evolutionarily conserved (Supplementary Fig. 9), suggesting that both the molecular basis of TACC–chTOG interaction and the assembly mechanism described here are also conserved.

**Figure 6:**
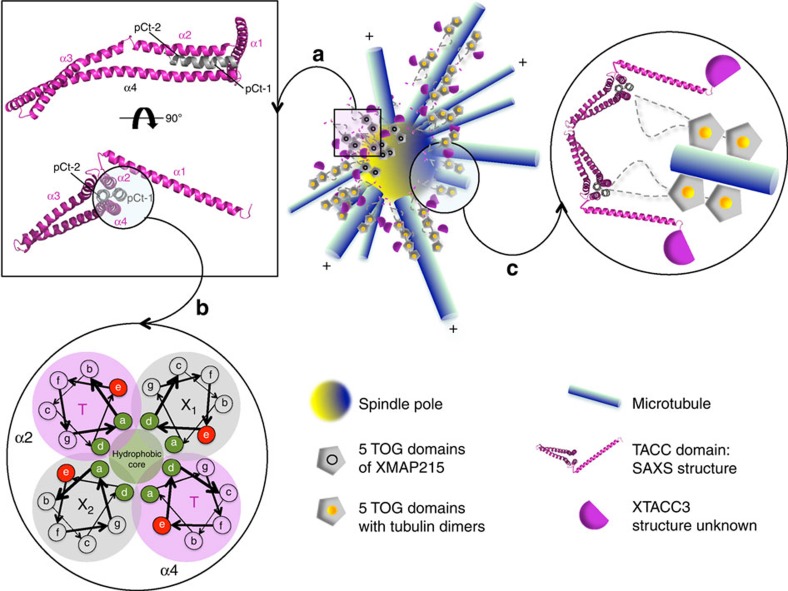
A model depicting XTACC3–XMAP215 interaction and MT elongation. (**a**) Model of the TD4–pCt interaction showing the SAXS structure of a TD monomer interaction with XMAP215 peptide (pCt) via the C-terminal coiled-coil association forming a four-helix bundle. The localization of the complex is steered by the TD. (**b**) A wheel diagram showing a possible association of the heptad repeats. The assembly would consist of a hetero-tetramer (X1 and X2- two molecules of XMAP215; T-TD, α2 and α4) forming an internal hydrophobic core, with the charged residues on the external surface. (**c**) A TD-directed recruitment of XMAP215 promotes MT growth whereby the TOG domains of XMAP215 add tubulin dimers processively.

The fact that TD induces oligomerization and that each domain bind two XMAP215 C-terminal peptides (Fig. 2c and Supplementary Fig. 5) suggests a mechanism whereby XTACC3 regulates XMAP215 through its efficient recruitment to the centrosome and spindle poles during mitosis. Thus, our study suggests a molecular mechanism for this efficient recruitment by showing that one molecule of TACC3 may interact with two molecules of XMAP215 forming a four-helix coiled-coil bundle (Fig. 6). Oligomerization of TACC3 could also be critical, as this would increase XTACC3 concentration locally. This will consequently result in the recruitment of large amounts of XMAP215, providing a mechanism that will efficiently favour the elongation of MT during spindle formation.

Recent study using XMAP215 mutants with different MT polymerase activities has shown that there is a direct correlation between the MT growth rate and spindle size^4^. Furthermore, increasing the concentration of XMAP215 in the egg extract results in proportionally longer spindles until a plateau is reached, thereby suggesting a saturation of XMAP215 binding sites^4^. These results indicate that the regulation of XMAP215 recruitment and/or activity is key for proper-sized spindle formation. In agreement with these data, we found that increasing XTACC3 concentrations in egg extract resulted in the formation of longer spindles in a XMAP215-dependent manner (Fig. 5e,f). Thus, the role of XTACC3 as XMAP215 regulator may be critical in this process.

Given the role of TACC and chTOG proteins in tumorigenic processes^22^,^23^ and its importance in cell division, any information of their role in cell cycle progression and centrosome maturation will provide novel features of its regulation. The structure and proposed mechanism may provide new therapeutic strategies against cancer by targeting this key TACC–chTOG interaction.

## Methods

### Molecular biology and protein purification

Various fragments of XTACC3 and XMAP215 were identified using controlled proteolysis and different coiled-coil prediction softwares. The TDs were cloned into pOPIN vectors using the In-Fusion PCR cloning method, a versatile ligation-independent cloning system engineered for high-throughput screening^24^. To aid protein overexpression and purification, two different pOPIN vectors were used in this study in order to generate fusion proteins with different tags, a His-GST tag or His-Sumo tag in pOPINJ and pOPINS, respectively. The XMAP215 domains were cloned into a pET vector with N-terminal His-tag. Various TDs were expressed in either *Escherichia coli* BL21(DE3) or Roseta pLysS at 37 °C. Cells were induced by the addition of 0.3 mM isopropyl-β-D-thiogalactopyranoside to a mid-log culture, and then cells were harvested either 3 h after induction or after 20 h by lowering the temperature to 22 °C. For double-labelled NMR samples (TD4 and XMAP-Ct), cells were grown in *E. coli* in minimal media by using ^15^NH_4_Cl and ^13^C-glucose as the sole nitrogen and carbon sources. Here cells were induced with isopropyl-β-D-thiogalactopyranoside at a higher density (OD_600_=0.8) and harvested after overnight expression at 37 °C. Recombinant proteins were purified from clarified crude cell extracts using a combination of immobilized metal ion affinity, GST column and gel filtration chromatography. The His-GST tag was removed by precision digest (3 h at room temperature), followed by NTA Ni-column chromatography, and the protein was further purified using a final size-exclusion column (Superdex 200, GE Healthcare) equilibrated in 20 mM Tris (pH 8.0), 300 mM NaCl and 0.25 mM TCEP, unless otherwise stated. Protein purity was monitored by SDS–polyacrylamide gel electrophoresis (SDS–PAGE) and electrospray ionization mass spectrometry. For double-labelled XMAP-Ct, the His-tag was not removed and the NMR samples were dialysed in 20 mM sodium phosphate pH 7.5, 150 mM NaCl, 0.25 mM TCEP, 90% H_2_O/10% D_2_O solution at pH 7.6. In all cases, the protein concentration was determined from the absorbance at 280 nm using a molar extinction coefficient derived by summing the contributions from tyrosine and tryptophan residues. His-XTACC3 full length and His-XTACC3-M4 were expressed and purified as in ref. 8. Aliquots of pure proteins were flash-frozen in liquid nitrogen and stored at −80 °C. PCR-mediated site-directed mutagenesis was performed using PfuTurbo DNA polymerase (Stratagene) according to the manufacturer’s protocol. All mutations were verified by DNA sequencing, and the list of constructs used in this study are shown in Supplementary Table 1.

### CD and thermal denaturation

Far ultraviolet CD, spectra (260–190 nm) were recorded using a Jasco J715 spectropolarimeter purged with nitrogen gas. Spectra were recorded in 0.1 cm cells at 20 °C in 20 mM Tris-HCl, pH 7.5, 150 mM NaCl, 0.25 mM TCEP at a protein concentration of 20–50 μg ml^−1^. Thermal denaturation studies by CD were recorded at 222 nm, with 10 °C increments, ranging from 10 to 90 °C. Protein folding data were fit as a two-state transition using the programme PRISM4.

### Isothermal calorimetry

Complex formation between TD4 and pCt was measured by ITC using a MicroCal Auto-IT200 microcalorimeter (GE Healthcare). Samples were dialysed into ITC buffer (20 mM HEPES, pH 7.5, 150 mM NaCl and 0.25 mM TCEP, pH 7.5). Typically, the sample cell contained TD4 (10–20 μM) and the syringe was filled with pCt (400–500 μM). Titrations with 20 injections of 10 μl were carried out at 25 °C. Heats of dilution were subtracted from the raw titration data before analysis. Data were fitted by least-square procedures assuming a one-site binding model using Microcal Origin version 7.0.

### Surface plasmon resonance

SPR assays were performed on a BIAcore X100 (GE Healthcare) instrument at 20 °C. Two different sets of experiments were carried out on two different CM5 sensor chip using an amine-coupling kit (GE Healthcare/BIAcore). In the first series of experiments, XMAP-Ct (50 μg ml^−1^) was immobilized on the CM5 chip and varying concentrations of TDs (TD4 or TD5, 10–250 μM) analytes were injected. In the second set of experiments, TD4 (50 μg ml^−1^) was the ligand coupled to the chip and various concentrations of peptide analytes (pCt or pCt_KD, 10–250 μM) were injected. In all cases, the analytes were flowed at a rate of 30 μl min^−1^ for 180 s to allow an association, followed by 180 s for dissociation over immobilized protein in HEPES-buffered saline (HBS) (100 mM HEPES, 150 mM NaCl and 0.05% P20, pH 7.4). The sensograms were subjected to global analysis using the BIA-evaluation software 2.0. A steady-state analysis using a non-linear least-square fitting, the equilibrium dissociation constant (*K*_D_) was determined by fitting the data to a single-site interaction model.

### Multi-angle light scattering

Molecular mass and molecular mass distributions were determined using online SEC-MALS. Samples were applied in a volume of 100 μl to a Superdex 200 10/300 GL column equilibrated in 20 mM Tris/HCl, 150 mM NaCl and 0.25 mM TCEP, pH 8.0, at a flow rate of 0.5 ml min^−1^. The column was mounted on a Jasco HPLC controlled by the Chrompass software package. The protein concentration of the eluent was determined from the RI(*n*) change (d*n*/d*c*=0.186, where *c* is solute concentration) using an OPTILAB-rEX differential refractometer equipped with a Peltier temperature-regulated flow cell, maintained at 25 °C (Wyatt Technology).

### Antibodies and western blot

Polyclonal rabbit antibodies anti-XTACC3 and anti-GST were produced in the laboratory against recombinant proteins, His-XTACC3 and GST, respectively. The 1C1 monoclonal anti-Eg2 antibody was a gift from C. Prigent (CNRS, Rennes, France). The 135 and 137 monoclonal anti-XTACC3 antibody was made in-house and generated by injecting mice with recombinant His-XTACC3. Blots were developed using Alexa Fluor 680 (Invitrogen) and IRdye 800CW (LI-COR) labelled antibodies, and were analysed through the Odyssey Infrared imaging system (LI-COR). Anti N-term-XMAP215 and C-term-XMAP215 were generated as in ref. 15.

### Xenopus egg extract preparation and pull-down experiments

Preparation of fresh cytosolic factor-arrested *Xenopus* egg extract (CSF extract), spindles and centrosomes asters is fully described in ref. 25. XTACC3 depletion experiments were performed by three successive rounds of XTACC3 immunoprecipitation^8^. Recombinant GST- and His-tagged proteins were then added to XTACC3 endogenous concentration (150 nM). Experiments with excess GST or XMAP-Ct, were performed by adding 15 μM recombinant protein. For GST pull-downs, 20 μl protein A-conjugated Dynabeads 280 (Invitrogen) were washed three times with 1 ml of PBS–Triton (0.1%) and then incubated for 30 min at room temperature on a rotating wheel with anti-GST antibodies. Antibody-coated beads were washed twice with 1 ml of PBS–Triton (0.1%) and twice with 1 ml of CSF-XB and then incubated for 60 min on ice with 60 μl of CSF-egg extract supplemented with 3 μM of recombinant protein previously incubated for 15 min at 20 °C. Beads were retrieved on a magnet for 10 min, washed twice with 0.5 ml of CSF-XB and twice with 0.5 ml of 0.1% PBS–Triton, both containing phosphatases inhibitors (100 mM NaF, 80 mM β-glycerophosphate and 1 mM Na_3_VO_3_). Proteins were eluted from the beads by incubating in SDS loading buffer for 10 min at room temperature before running a SDS–PAGE and analysed by western blot. Alternatively, antibody-coated beads were incubated for 90 min at 4 °C on rotating wheel with 0.2 μM of GST proteins in 200 μl of 0.1% PBS–Triton+BSA 0.1 mg ml^−1^. Beads were then washed twice with 1 ml of PBS–Triton 0.1% and twice with 1 ml of CSF-XB, followed by re-suspending in 40 μl of CSF extract, incubated at 20 °C for 15 min, then incubated on ice for 60 min and finally retrieved as previously described. His-tag pull-downs were performed as described above but using: Ni-NTA agarose beads (Qiagen). Recombinant proteins were added to extract at a final concentration of 0.5 μM or loaded on the beads at a final concentration of 0.4 μM.

### Immunofluorescence and quantifications

Spindles and asters assembled in egg extract were processed for immunofluorescence as described^26^. Samples were visualized with a × 40 objective on an inverted DMI-6000 Leica wide-field fluorescent microscope. Images were processed and mounted with Adobe Photoshop CS5.1. Spindle length and area, and tubulin, XTACC3 and XMAP215 intensities were calculated with ImageJ (http://rsbweb.nih.gov/ij/docs/index.html) using a homemade macro (available from I.V. upon request). In brief, image threshold was 10% of the maximum intensity of the image and the ‘analyse particles showing ellipses’ function of ImageJ was used to detect each spindle. Each detected particle was transferred at the centre of a new image with black background, orienting it with the poles on the horizontal axis. Each generated image was manually selected in order to discard detection errors. Next, for each image, a threshold was set on a custom-selected percentage of the average intensity of the image (the percentage used changed from experiment to experiment, depending on the tubulin signal). Again, the ‘analyse particles showing ellipses’ function of ImageJ was used to define the ROI of the single spindle in the image. The length and the area of the detected ROI were used as a measurement of spindle length and area. Average intensity of the different channels within the ROI was considered as the measurement of the average intensity of the fluorophore of interest. Values were then analysed with Excel (Microsoft). Reported values corresponded to the normalized weighted average of >30 spindles per condition in three independent experiments (two in the case of XMAP215 density).

### NMR spectra acquisition, analysis and assignment

^13^C, ^15^N XMAP-Ct-labelled samples for NMR were prepared at 0.2–0.5 mM in 20 mM sodium phosphate buffer, 150 mM NaCl and 0.25 mM TCEP at pH 5.9, 7.0 and 7.5, respectively. The ^13^C, ^15^N TD4-XMAP-Ct complex was prepared in 20 mM Tris buffer, 150 mM NaCl, 0.1% LDAO (N,N-dimethyldodecylamine-N-oxide), 0.25 mM TCEP buffer, pH 7.0 to a concentration 0.4 mM. XMAP215 peptide (pCt) samples were of 0.3–0.4 mM in 20 mM sodium phosphate buffer, 150 mM NaCl, 0.25 mM TCEP, pH 6.1 (pCt), pH 5.5 (pCt_KD), pH 6.2 (pCt_LS). All NMR samples contained 10% (vol) D_2_O and sodium 4,4-dimethyl-4-silapentane-1-sulfonate as the chemical shift reference. NMR experiments for protein samples were run at 25 and at 5 °C, and with peptides at 25 °C. Spectra were acquired on a Bruker Avance 800 MHz spectrometer equipped with a z-gradient cryoprobe. Standard pulse sequences corresponding to 2D ^15^N-HSQC, 2D ^13^C-HSQC, 3D HNCA, 3D HN(CO)CA, 3D CBCANH and 3D CBCA(CO)NH spectra were acquired and analysed for the complete assignment of the backbone of XMAP_Ct at pH 5.9, 7.0 and 7.5. To enable partial assignment of TD4, conventional 3D HN(CO)CA, CBCA(CO)NH, CBCANH, HNC-TOCSY and HNN-TOCSY spectra were recorded. All assignments were confirmed using a set of β- and γ-carbon edited ^1^H–^15^N spectra^27^. For XMAP-Ct peptides, 2D TOCSY (60 ms mixing time), NOESY (150 ms mixing time) and natural abundance ^13^C-HSQC spectra were acquired and assigned following standard methods.

### NMR-monitored titration experiments

Increasing amounts of lyophilized XMAP-Ct were added to a ^13^C, ^15^N TD4 sample in 20 mM sodium phosphate, 150 mM NaCl, 0.25 mM TCEP, 0.1% LDAO, pH 7.0 buffer and a series of ^15^N-HSQC spectra were recorded at each titration point. The XMAP-Ct interaction with TD4 was also followed by comparing the ^15^N-HSQC spectrum of the free ^13^C, ^15^N XMAP-Ct at pH 7.5 with that obtained after the addition of unlabelled TD4 stock solution to an approximate protein:protein ratio of 1:1. Changes of peak intensity and position were monitored, and both the chemical shift and line width changes were analysed. In all cases, the pH was checked at the starting and final points of the titrations. The spectra were processed with Bruker Topspin (Bruker, Germany) and spectral analysis was performed with Sparky 3. (Goddard TD, Kneller DG, 2005, Sparky 3, University of California, San Francisco).

### NMR estimation of helix populations

Helix populations were quantified from the ^13^Cα or ^1^Hα conformational chemical shift values as described^28^. The random coil values were obtained from ref. 29.

### SAXS measurements and analysis

The SAXS measurements were carried out on an instrument optimized for solution scattering and is the prototype of the commercially available NanoSTAR instrument from Bruker AXS^30^ at Aarhus University. The instrument uses a rotating anode as source (Cu Kα) and a HiSTAR gas detector. It was recently modified to use home-build ‘scatterless’ slits^31^ and to include a home-build flow-through quarts capillary (thermo stated) for fast sample change. The data are given as a function of the scattering vector modulus *q*=4*π* sin*θ*)/*λ*, where 2*θ* is the scattering angle and *λ* is the X-ray wavelength. Initial data treatment, background subtractions and absolute calibration using water as a primary standard were done using the SUPERSAXS programme package (C.L.P. Oliveria and J.S. Pedersen, unpublished observations). Samples and buffers were measured at 4 °C. The sample concentrations were ~1–4 mg ml^−1^ for the wild-type and TD4-M2 samples.

Protein in the absence of peptide (pCt) agreed with a reduced *χ*^2^ (=1.55), presenting only small differences between the two data sets. The corresponding comparison between samples with peptide using a least-square approach gives a reduced *χ*^2^=1.95 again showing little difference. The data was further analysed by indirect Fourier transformation^32^, which provides the pair distance distribution function *p*(*r*), from which the forward scattering *I*(*q*=0) and the radius of gyration *R*_g_ were determined. The *p*(*r*) function is a histogram of distances between pairs of points within the particles weighted by the excess electron density at the points and this is shown in Fig. 3a. The functions are similar with subtle differences between samples with and without polypeptides. All functions agree with a maximum diameter of the particles of 300 Å and a shape suggesting an elongated rod-like shape^33^. The results for the molecular mass derived from the forward scattering using a standard contrast factor of Δ*ρ*_m_=2.00 × 10^−10^ cm g^−1^ and the equation *M*=*I*(0)/(*c* Δ*ρ*_m_^2^), where *c* is the concentration, and the radius of gyration are displayed in Supplementary Table 2.

SAXS measurements of TD4 were performed at three different protein concentrations (~4, 2 and 1 mg ml^−1^) in combination with or without pCt. Scattering data from various samples as a function of the modulus of the scattering vector, *q*, were scaled to the highest protein concentration to show small differences at low *q*. The data display a large range with approximate 1/*q* behaviour expected for rod-like particles. Given the concentration-dependent self-association behaviour of the TDs, we re-examined the oligomerization state of TD4 by MALS, and the experiment confirmed that TD4 at 1.5 mg ml^−1^ is a dimer (Supplementary Fig. 5c) and further SAXS data analysis was carried out at this concentration. TD4 was further analysed by an *ab initio* approach using the programme GASBOR (ref. 34). Dimers with P2 symmetry were generated and the structures were aligned by SUPCOMB (ref. 35). The procedures of GASBOR are based on simulated annealing optimization resulting in slightly different structures at each run. The structures can be considered to be identical within the resolution of the SAXS experiment. The models agree perfectly with the respective scattering data. Finally, a detailed model was built using a protein model of TD4 obtained using I-Tasser (ref. 17) as a starting point. A dimer model with contact in the loop region between helices α3 and α4, where the two molecules can form a contact with good match between the residues, was constructed. However, the model did not provide a satisfactory fit to the SAXS data and it was too small (200 Å) compared with the estimated maximum diameter (300 Å). Therefore, the helices at the two ends of the dimer (α1) were manually moved using the programme MASSHA (ref. 36), so that they are directed away from the rest of the molecule, extending its overall size. Using the programme CRYSOL (ref. 37), a comparison was made with the SAXS data and a reasonably good fit was obtained. The model was further optimized by rigid body refinements using the programme CORAL (ref. 38), optimizing the connections between the two parts of the dimer as well as the connections to the helices at the end of the model. A distance restraint was introduced so that residue 136 of the I-Tasser^17^ structure was within 7 Å of the corresponding residue on the other molecule. Initially, no symmetry was imposed on the model, however, in further runs, P2 symmetry was applied. For each of the cases with or without applied symmetry, 10 independent runs were performed and the models were aligned and compared. With P2 symmetry, they are almost identical, whereas there are some variations in the direction of the α1 helix without symmetry, demonstrating that the models are very robust. We note that for all runs, the models fitted the data very well with similar fit qualities, and thus the SAXS data do not allow us to distinguish between the two models. SAXS-derived models are available upon request.

## Author contributions

G.B.M., I.V. and G.M. conceived the study; G.B.M., T.C. and D.H. designed the constructs, G.B.M and J.G.P. expressed and isolated the proteins; M.F.G.-M. and M.B. performed and interpreted the NMR experiments; T.C., G.B.M. and I.P. performed the cell extracts and cell biology experiments; G.B.M., J.L. and J.S.P. performed the SAXS experiments; G.B.M., N.M. and F.B. performed the different biophysical experiments; and G.B.M., I.V. and G.M. wrote the paper.

## Additional information

**How to cite this article:** Mortuza, G. B. *et al*. XTACC3–XMAP215 association reveals an asymmetric interaction promoting microtubule elongation. *Nat. Commun.* 5:5072 doi: 10.1038/ncomms6072 (2014).

## Supplementary Material

Supplementary Information Supplementary Figures 1-9 and Supplementary Tables 1-2
