# Supplementary material for: XTACC3–XMAP215 association reveals an asymmetric interaction promoting microtubule elongation
Source: Nat Commun. 2014 Sep 29;5:5072. doi: 10.1038/ncomms6072 (PMC4200520; doi:10.1038/ncomms6072)
Supplement: Supplementary Information — Supplementary Figures 1-9 and Supplementary Tables 1-2 [file ncomms6072-s1.pdf]

Supplementary Fig1

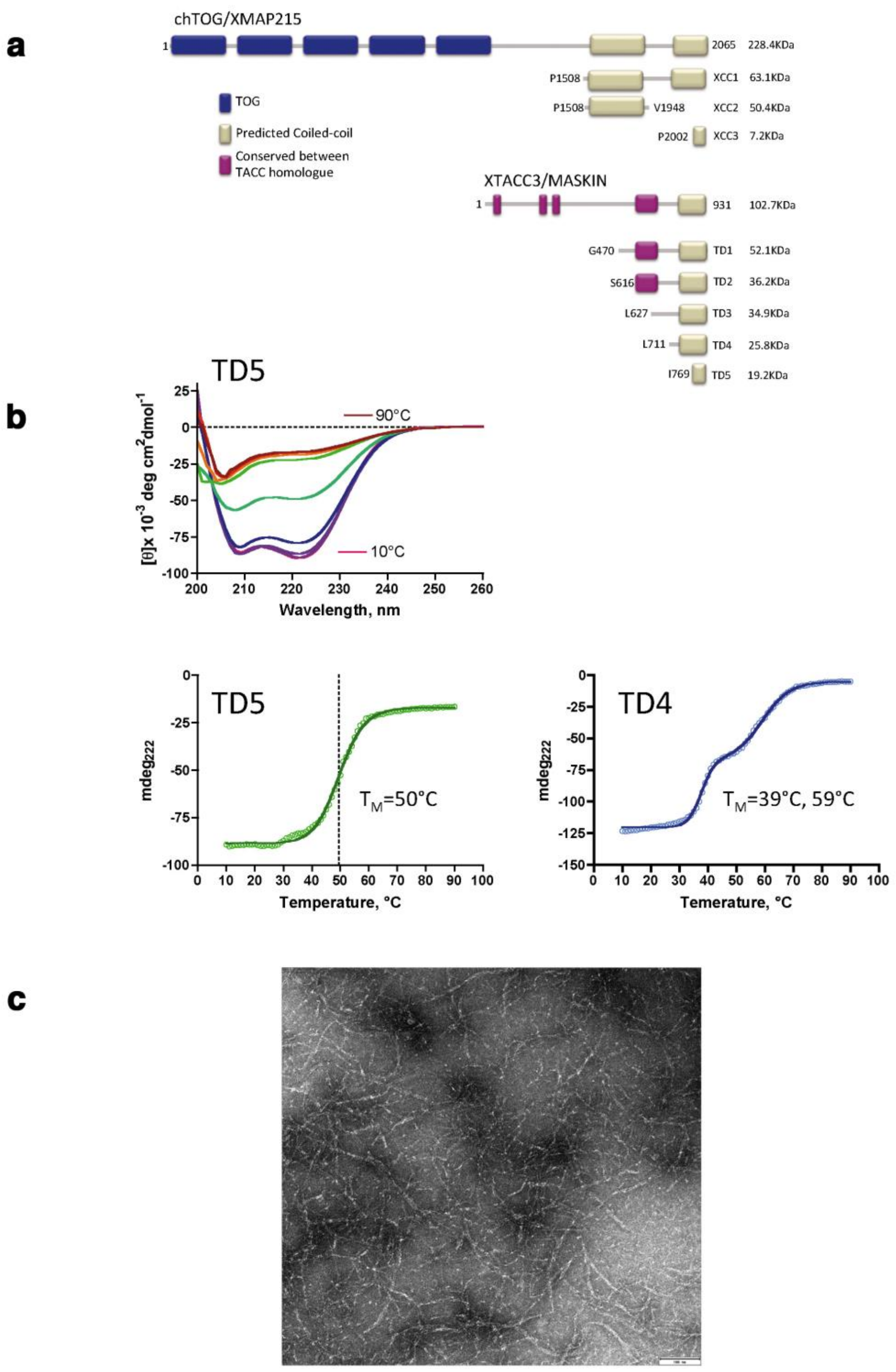

Supplementatry Fig 1. Domain structure, biophysical characterisation and electron microscopy of a TD. **(a)** XTACC3/Maskin and XMAP215/chTOG domain architecture. Various C-terminal fragments were cloned and expressed for biophysical and functional analysis. Regions conserved between TACC homologues are coloured in magenta and the coiled coil regions in grey. **(b)** Thermal denaturation of the TDs (TD4 and 5) monitored by circular dichroism (CD) showing different unfolding transitions. **(c)** Negative-stained electron micrograph showing TD4 filaments. The white bar represents 100 [illegible].

Supplementary Fig 2

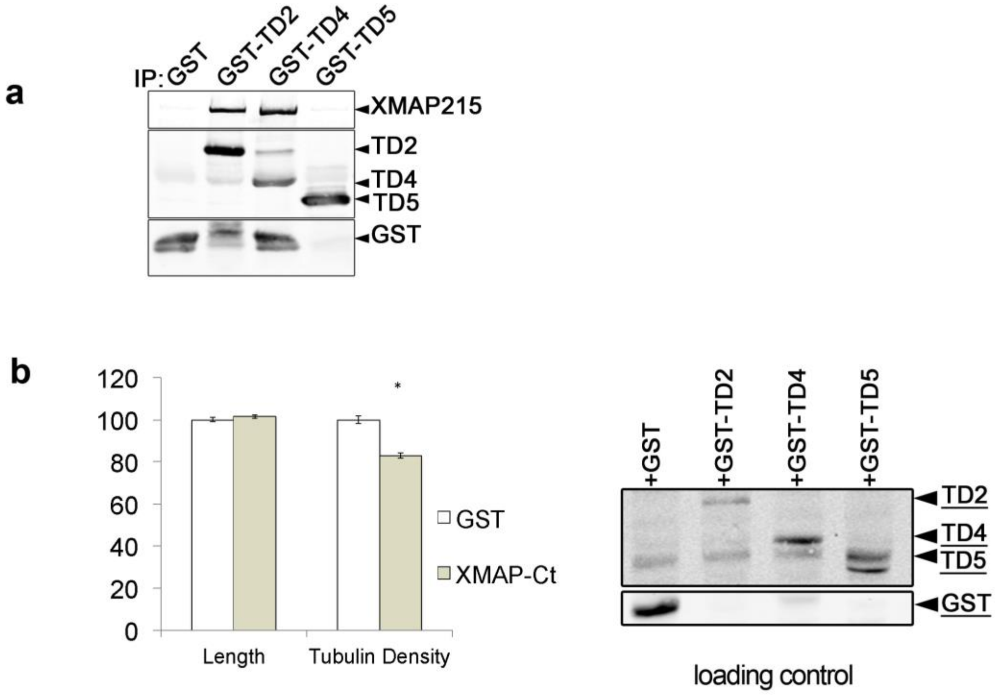

Supplementary Fig 2. Functional analysis of the minimal TD domain of XTACC3 (a) Western blot analysis of anti-GST pull-downs from egg extracts containing GST, GST-TD2, GST-TD4 or GST-TD5 (as indicated on top). The blot was probed with an anti-XMAP215 (upper row) showing that GST-TD2 and GST-TD4 can pull-down endogenous XMAP215, whereas GST-TD5 cannot. The different GST-TD fusion proteins were visualized using an anti-GST antibody. (**b)** Bar chart showing spindle length, tubulin density and XTACC3 localization normalized on tubulin density. All values are the weighted mean of three independent experiments. Although spindles assembled in the presence of XMAP-Ct display similar lengths to controls, their tubulin density is reduced by 20% and the recruitment of XTACC3 is also reduced. (*, p<0,01). Loading control. Western blot analysis of the egg extracts used to assemble the spindles represented in Fig1c. The blot was examined using anti-GST antibodies.

Supplementary Fig 3

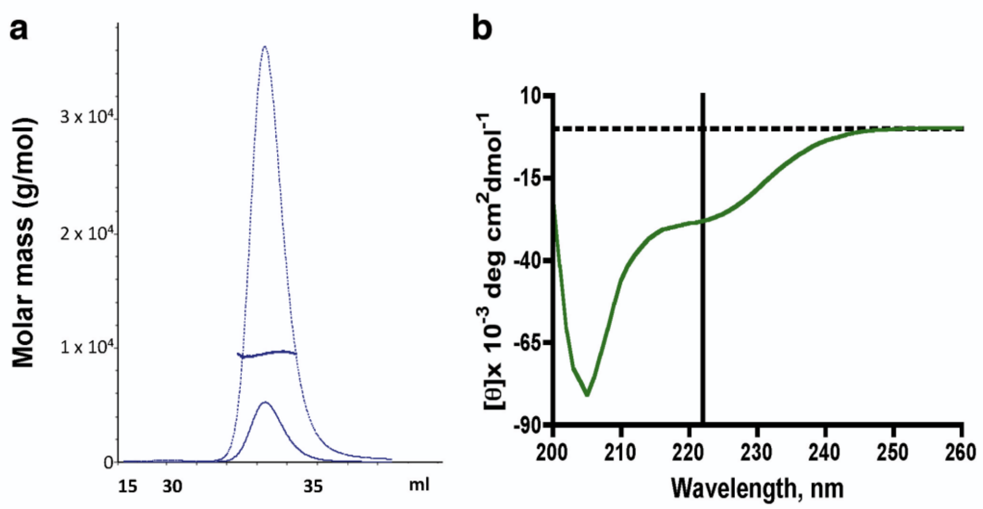

Supplementary Fig 3. Multi-angle light scattering (MALS) and CD of XMAP-Ct. (**a)** MALS elution profile of XMAP-Ct showing that it behaves as a monomer at 7 mg/ml (9.5 kDa MW). (**b)** Circular dichroism spectrum showing almost no secondary structural elements of this XMAP215 fragment.

Supplementary Fig 4

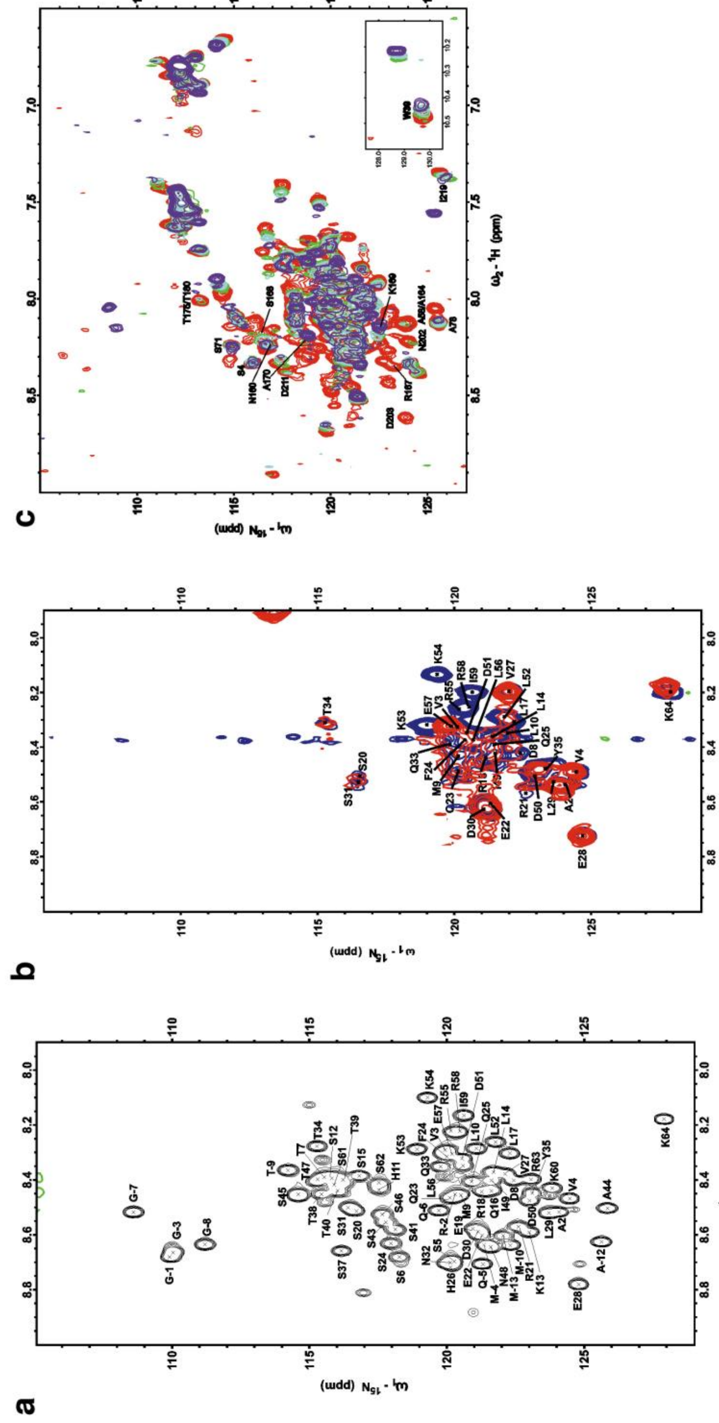

Supplementary Fig 4. NMR data analysis showing the regions involved in XMAP-Ct-TD4 interaction. (a) Assigned ^{15}N-HSQC spectrum of XMAP-Ct at pH 5.9. Signals corresponding to the H-N amide groups are labelled with the one letter code and the sequence number. Negative numbers correspond to the His-tag sequence at the N-term. (**b)** The same region of the ^{15}N-HSQC of free XMAP-Ct (blue contours) and XMAP-Ct:TD4 complex (red contours) at pH 7.5. Signals corresponding to non-exchanged H-N amide groups at this pH are labelled with the one letter code and the sequence number. (**c)** NMR data of TD4-pCt interaction at pH 7.0 Superposition of ^{15}N-HSQC spectra of TD4 free (red) and in the presence of different amounts of pCt, which would correspond to estimated TD4:pCt ratios of 1:25 (green), 1:35 (cyan), and 1:55 (purple). Selected assigned residues are labelled with the one code letter and the residue number. In the case of ambiguity (T175/T180; A58/A164), both possibilities are shown.

Supplementary Fig 5

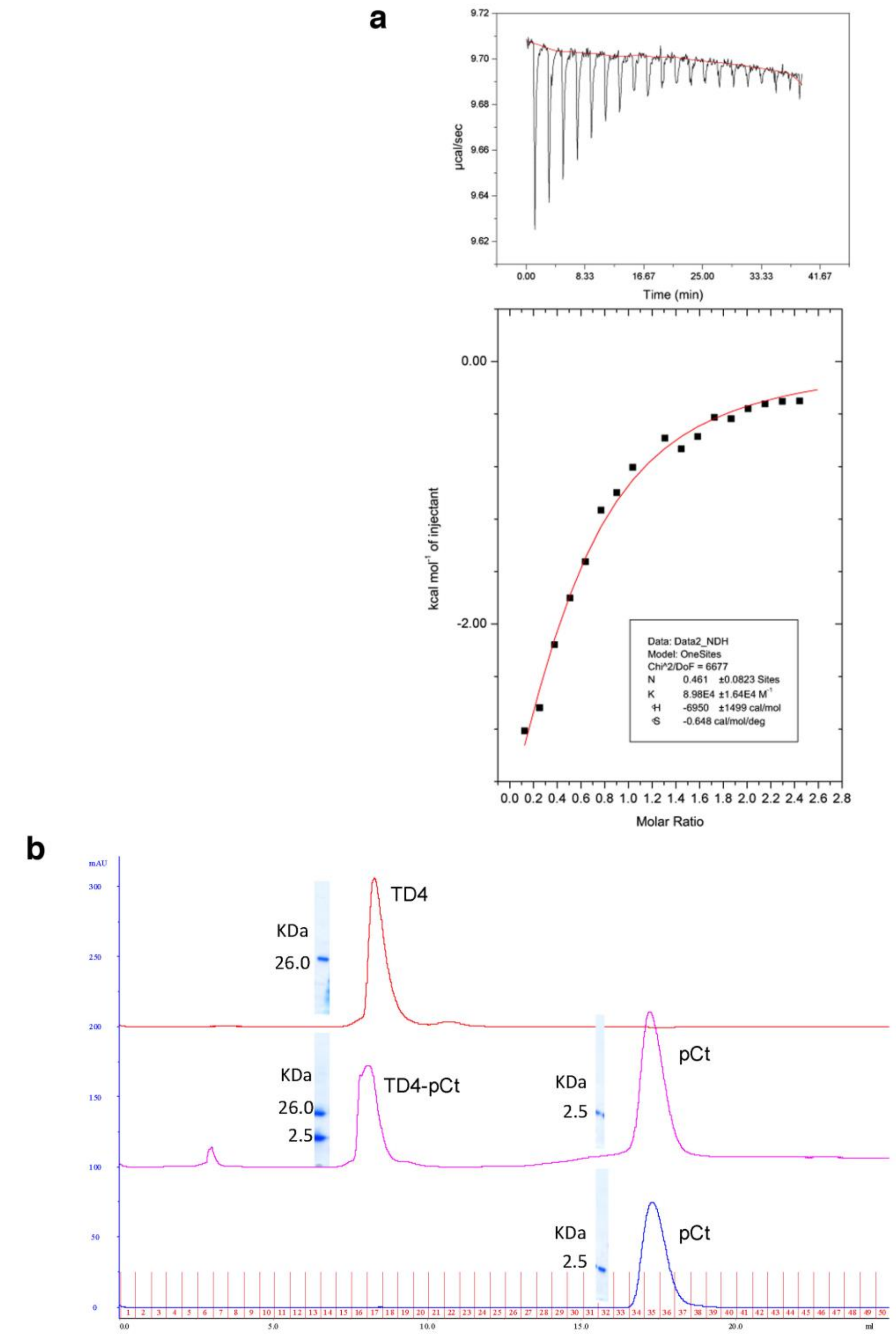

Supplementary Fig 5. TD4, pCt binding and MALS analysis. (a) Isothermal Titration Calorimetry (ITC) curve of pCt binding to TD4. **(b)** Size exclusion chromatography shows that pCt elution profile is shifted in the presence of TD4, indicating that the peptide binds the TACC domain.

Supplementary Fig5 cont.

c

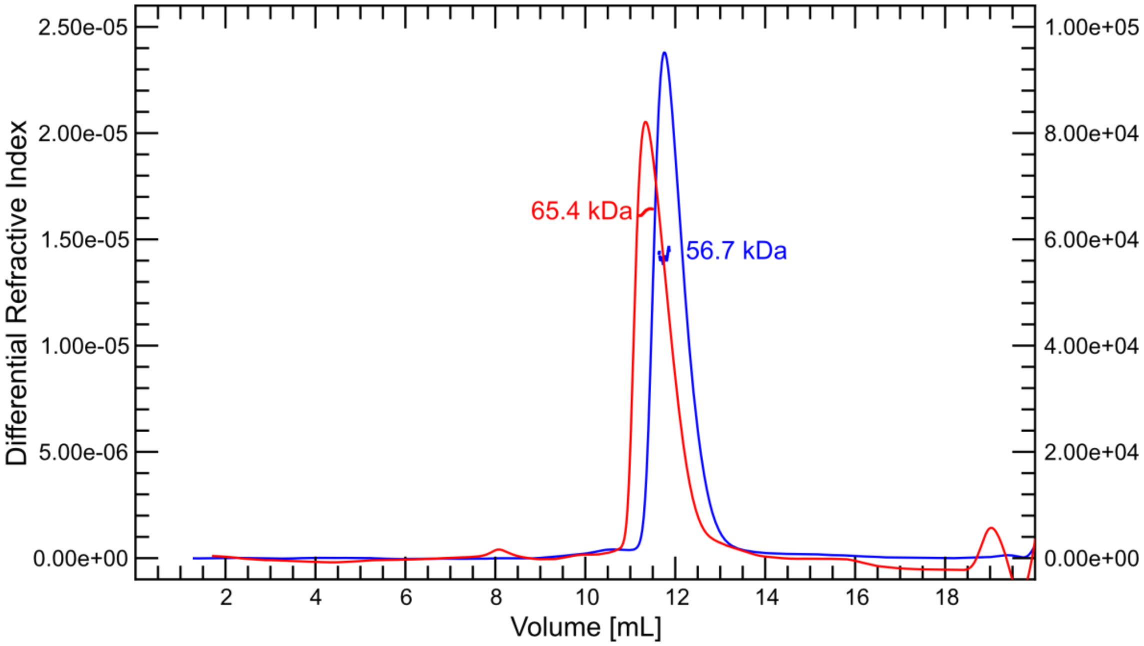

Supplementary Fig 5. **TD4, pCt binding and MALS analysis**. **(c)** MALS of TD4 (blue) and the TD4:pCt complex (red). The TD4 run reveals that this domain is a dimer (56.7 kDa) at the concentration used for the SAXS experiments. The MALS run of the TD4:pCt complex (65.4 kDa) shows a shift of approximately 10 kDa, suggesting that 4 peptides can bind the TD4 dimer.

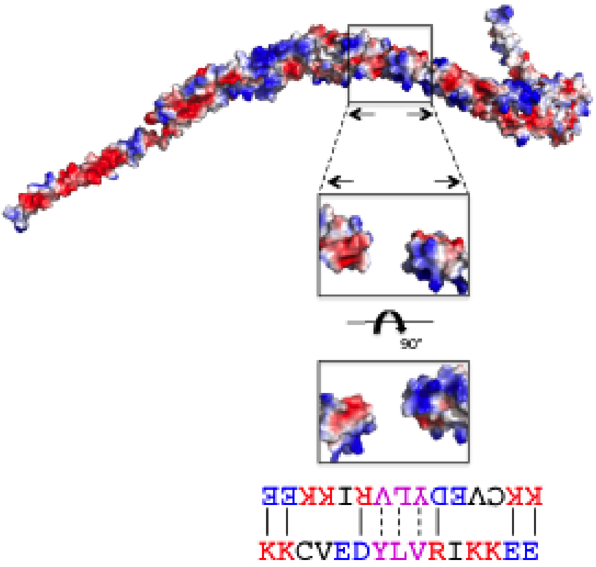

Supplementary Fig 6. TD4 and dimerization interface mutants. Surface representation of TD4 dimer showing the protein electrostatic potential and the dimer interface. The residues at the dimer interface are coloured according to their electrostatic potential. The solid and dashed lines represent the possible polar and hydrophobic contacts.

Supplementary Fig 7

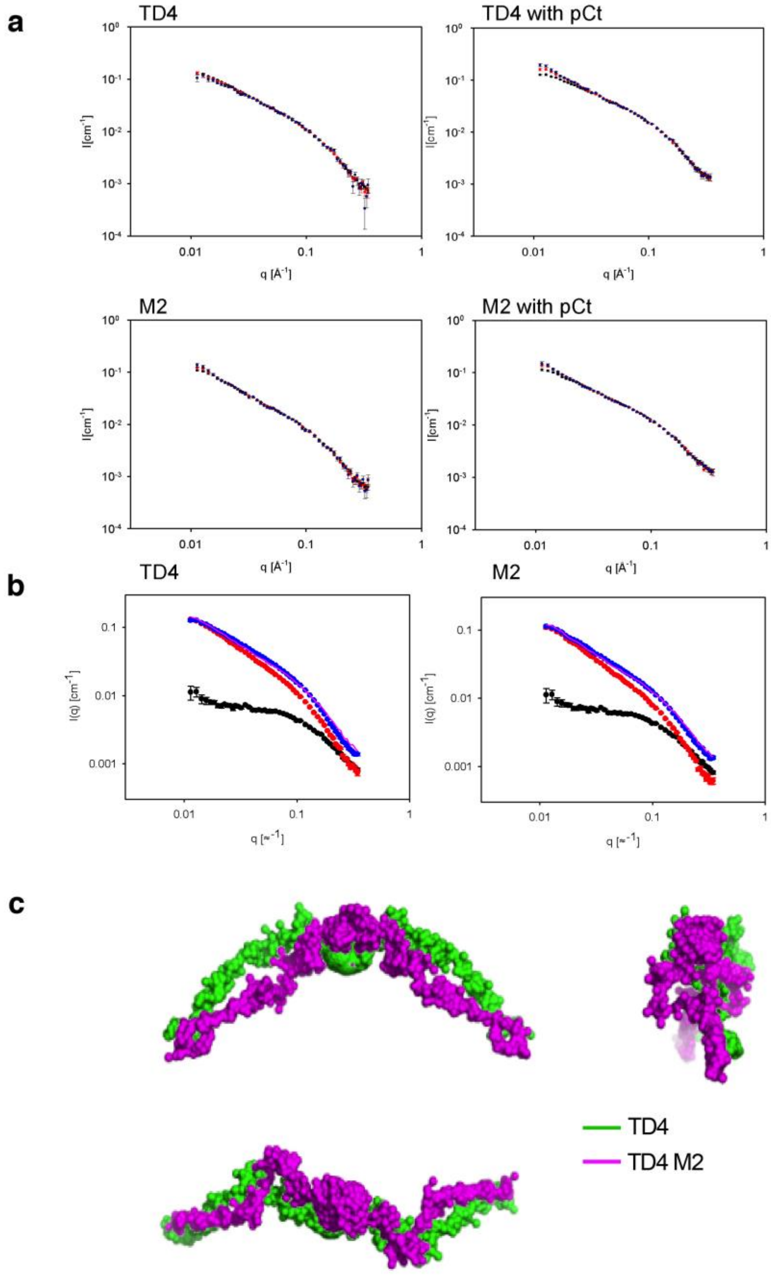

Supplementary Fig 7 SAXS data of TD4 and TD4-M2 mutant with and without pCt. To discard any effect arising from changes in TD4 structure we used SAXS to analyse TD4-M2 and test whether there is any dramatic structural reorganization that might alter pCt binding. (**a**) SAXS data for the various samples was scaled to the highest concentration (about 4 mg/ml, black points) using a least-squares approach. Curves in red are for approximately 2 mg/ml and those in blue are approximately 1 mg/ml. (**b**) SAXS data shown for peptide (black), protein without peptide (red), protein with peptide (blue), and linear combination of the scattering from peptide and protein without peptide (cyan line) as obtained from a least-squares fit. The scale factor of the peptide contribution for TD4-M2 was similar to that for the wt protein (0.95+/-0.02). The linear combination does not fit satisfactory the SAXS data of the samples with TD4 and TD4-M2 with peptide, which demonstrates that complexes are formed. The reduced chi-square value of the fit of the linear combination to the scattering from the wild type with peptide is $\chi^2 = 41$, whereas for TD4-M2 it is $\chi^2 = 15$, which probably

demonstrates a more pronounced pCt interaction for wild type than for TD4-M2. (**c)** The SAXS data of TD4 and TD4-M2 were also analyzed by an *ab initio* approach for obtaining a first estimate of the structure. The *ab initio* models for dimers with P2 symmetry generated by GASBOR are shown and they are elongated structures that can be considered identical within the resolution of SAXS: TD4 (Green); TD4- M2 (magenta). The models have also been turned 90° around two perpendicular axes to show three different views.

Supplementary Fig 8

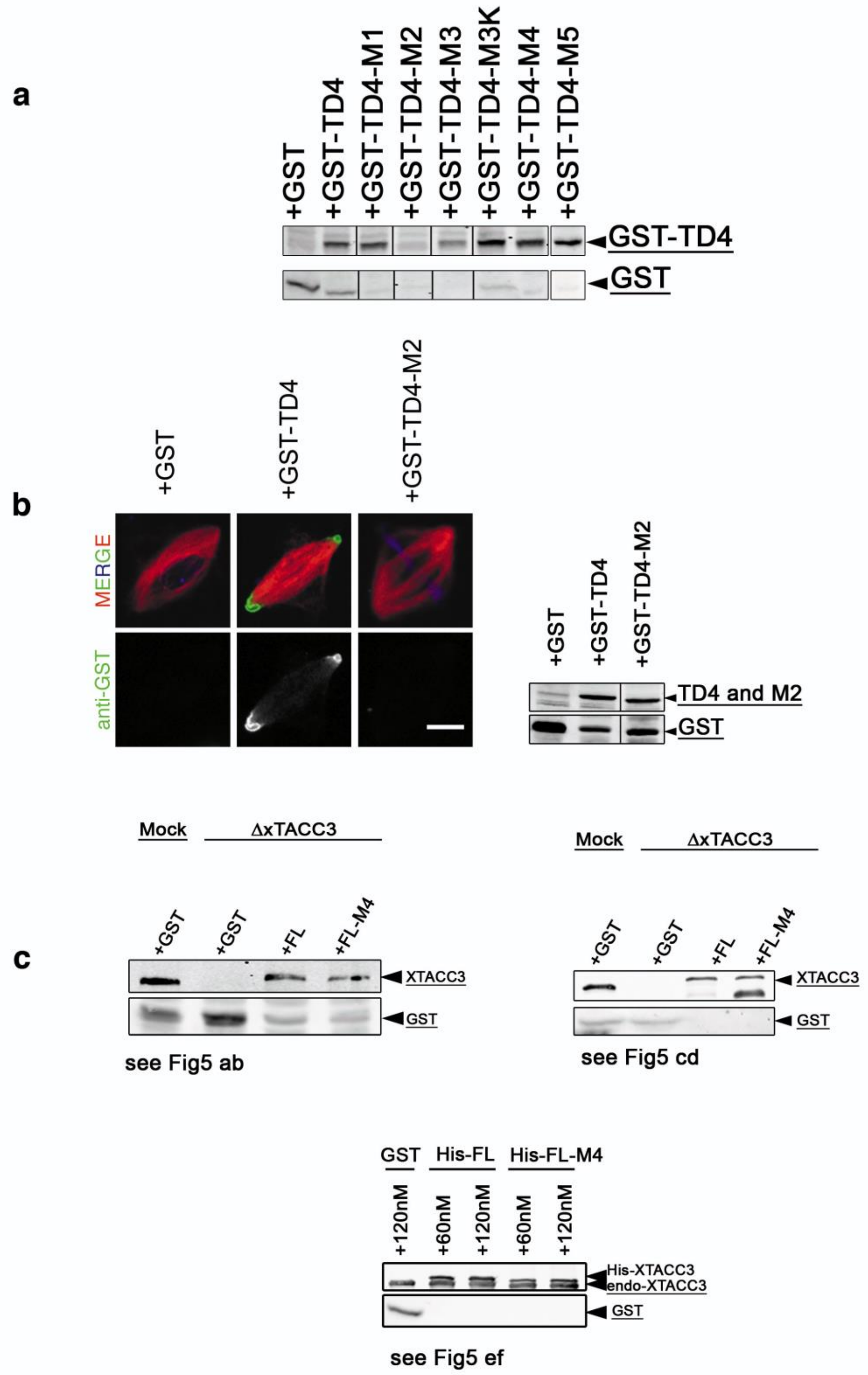

Supplementary Fig8. Loading controls. (**a**) Western blot analysis of the egg extracts used to assemble the spindles represented in Fig.4c was examined using anti-GST antibodies. (**b)** Representative images of spindles assembled in egg extracts supplemented with GST, GST-TD4 or GST-TD4-M2 as indicated. Samples were processed for IF using anti-GST antibodies. Even at high concentrations GST-TD4-M2 does not localize to the spindle. Scale bar 10μm (left). Western blot analysis of the egg extracts used to assemble the spindles represented in Supp. Fig. 7b. **(c)** Analysis of the egg extracts used to assemble the spindles represented in Fig.7a. The blot was examined using anti-GST and anti-XTACC3 antibodies. **(d)** Western blot analysis of the egg extracts used to assemble the spindles represented in Fig.5c. The blot was examined using anti-GST and anti-XTACC3 antibodies. **(e)** Western blot analysis of the egg extracts used to assemble the spindles represented in Fig 5E. The blot was examined using anti-GST and anti-XTACC3 antibodies.

Supplementary Fig9

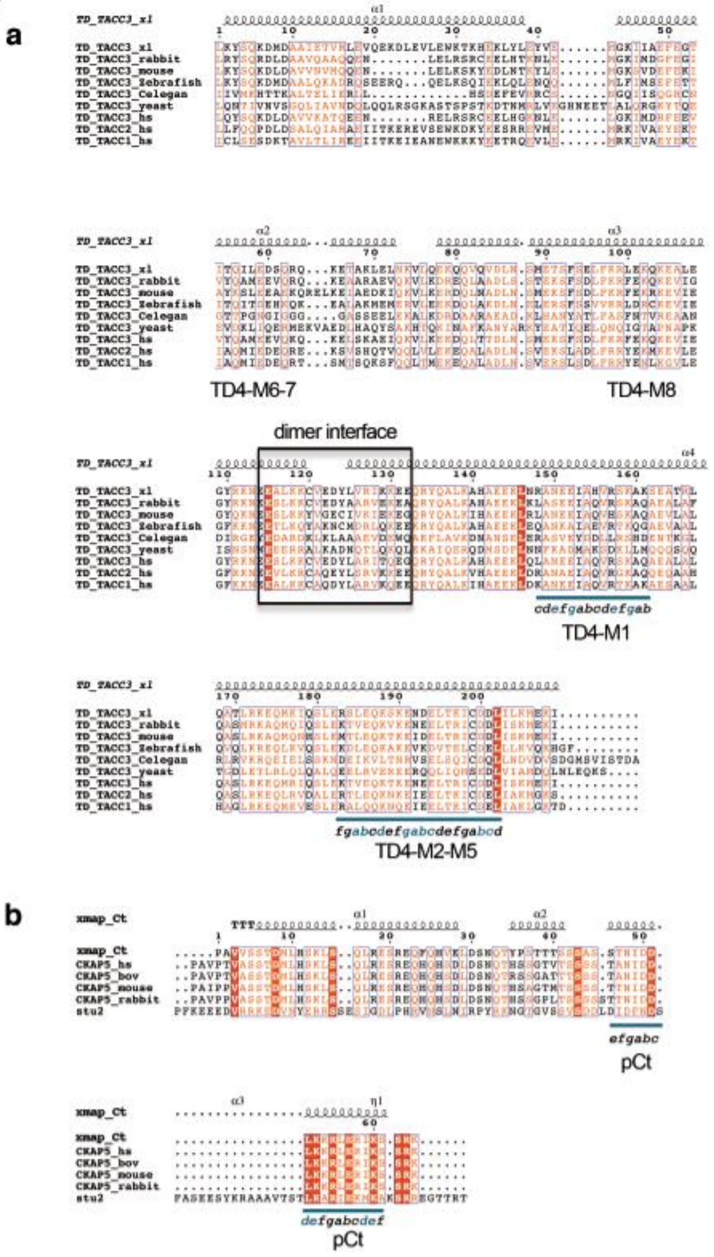

Supplementary Fig 9. Protein sequence alignment of the C-terminal regions of XTACC3 and XMAP215. **(a)** Sequence alignment of TD from various organisms together with the three different TACCs in human, showing a highly conserved dimer interface and heptad regions were mutations were performed. **(b)** Sequence alignment of XMAP215 (XMAP-Ct) with various vertebrates and yeast (Stu2) proteins showing a highly conserved C-terminal region, particularly in the ultimate 20 residues (pCt). The location of the dimer interface and regions of mutations performed on the TD are also highlighted The organisms (x.l) -Xenopus leavis; (m.m) – Mus musculus; (o.c) – Oryctolagous cunuculus; (c.e) - Caenorhabditis elegans; (s.p) - Schizosacharomyces pombe; (b.t) - Bos taurus; (h.s) - Homo sapeins.

Supp. Table 1.- *Nomenclature defining the different constructs and mutants used in this study*

Protein	Construct name	Residues
XTACC3	TD1	G470-I931
	TD2	S616-I931
	TD3	L627-I931
	TD4	L711-I931
	TD5	L769-I931
XMAP215	XMAP_Ct	P2002-K2065

XMAP215	Mutations	Heptad position
pCt	-	
pCt _KD	K2054D, K2061D	e, e
pCt _LS	L2053S, I2060S	d, d
XMAP_Ct		
XMAP_Ct _KD	K2054D, K2061D	e, e
XMAP_Ct _KR	K2054R, K2061R	e, e
XMAP_Ct _LI	L2053I, I2060L	d, d
XMAP_Ct _LS	L2053S, I2060S	d, d

TD mutants	Mutations	Heptad position
TD4-M1	N872A, E874A, R879A, K881A	e, g, e, g
TD4-M2	L907A, E908A, K910A, E913A, N914A	a, b, d, g, a
TD4-M3	D915A, E916A	b, c
TD4-M3K	D915K	b
TD4-M4	D922A, D923A	b, c
TD4-M5	K919A	f
TD4-M6	F772S, E773K, T775G, I776S, T777K, Q778G, S780S, E781G	d, e, c, d, e, f, a, b,
TD4-M7	F772G, E773A, T775G, I776A, T777A, Q778G, S780A, E781G	d, e, c, d, e, f, a, b,
TD4-M8	K824K, STOP	

Supp. Table 2.- *SAXS data of TD4 and TD4-M2 (see Supp. Table 1) in the presence and absence of pCt*

	**TD4 (mg/ml)**			**TD4+pCt (mg/ml)**			**TD4-M2 (mg/ml)**			**TD4-M2+pCt (mg/ml)**		
**Sample Conc.**	4.05	1.67	0.77	4.05	1.87	0.81	3.41	1.58	0.75	3.41	1.61	0.78
**I(0) [cm $^{-1}$]**	0.177 ±0.005	0.069 ±0.003	0.027 ±0.003	0.167 ±0.005	0.101 ±0.003	0.154 ±0.005	0.150 ±0.003	0.081 ±0.003	0.041 ±0.002	0.143 ±0.005	0.086 ±0.003	0.047 ±0.003
**M [kDa]**	65.8 ±1.9	62.3 ±2.4	52.6 ±6.5	62.1 ±1.9	81.8 ±3.2	106.5 ±6.6	62.2 ±2.0	77.2 ±2.5	82.7 ±4.0	63.2 ±2.0	80.4 ±2.5	90.8 ±6.4
**R_g [Å]**	87.1 ±2.0	86.3 ±4.0	78.4 ±8.0	80.1 ±3.0	86.3 ±3.0	98.7 ±3.0	91.5 ±2.0	97.8 ±2.0	100.2 ±3.0	83.3 ±3.0	91.6 ±3.0	98.6 ±4.0

Note that the values of the forward scattering I(0) and thus the values of the of the molecular mass M, as well as the values of the radius of gyration R_g are very sensitive to the background subtraction for the data points at the lowest q. Since the background subtraction is more critical for the low concentration samples, the values are less reliable for the diluted samples
